# Supplementary material for: Unravelling the interplay of nitrogen nutrition and the Botrytis cinerea pectin lyase BcPNL1 in modulating Arabidopsis thaliana susceptibility
Source: Commun Biol. 2025 Feb 8;8:204. doi: 10.1038/s42003-025-07642-7 (PMC11807159; doi:10.1038/s42003-025-07642-7)
Supplement: Supplementary file 1 — Supplementary Information [file 42003_2025_7642_MOESM1_ESM.pdf]

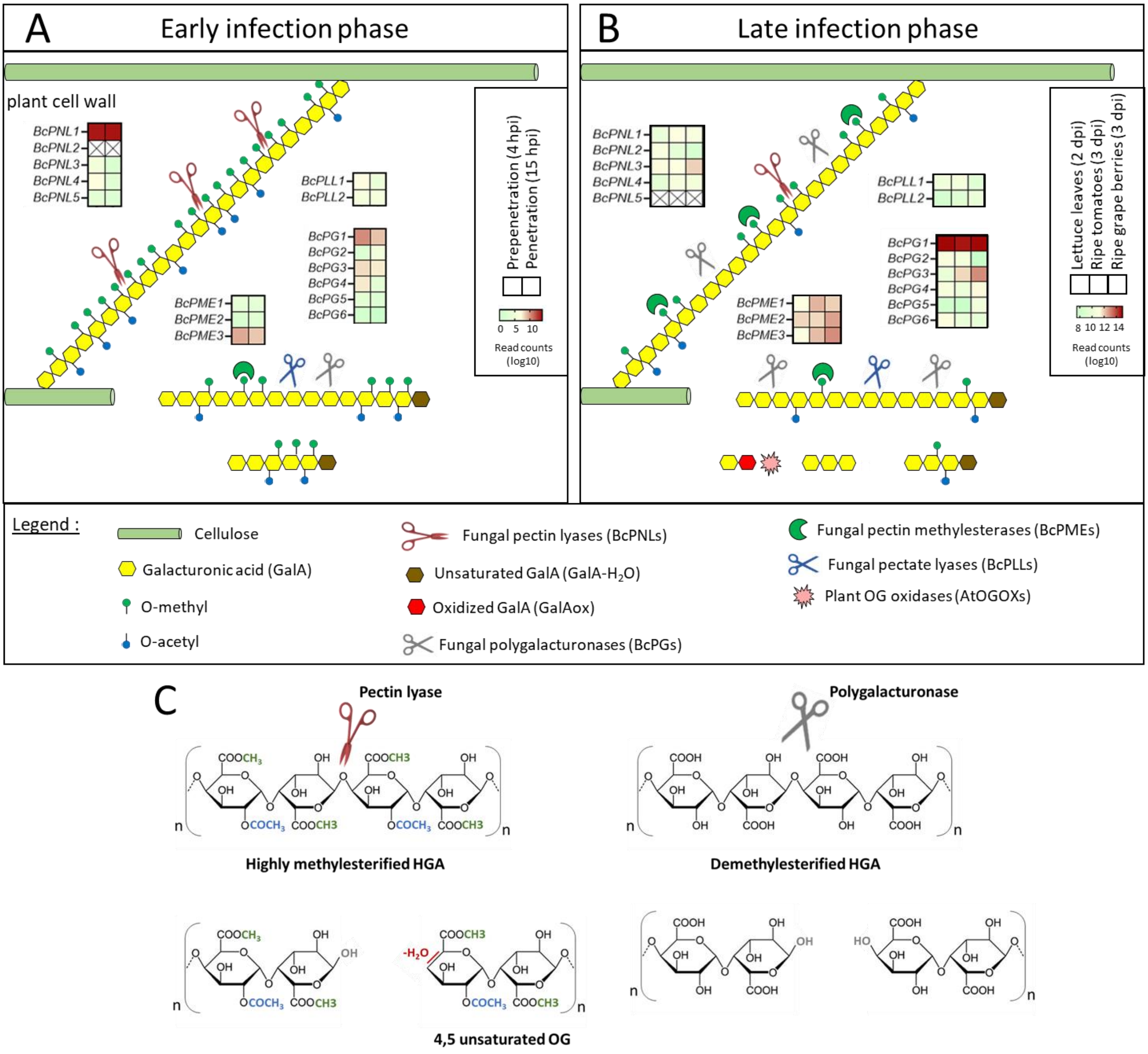

Supplementary Figure 1: The pectin-degrading machinery of B. cinerea at the early and late stages of the infection process. *This model is based on transcriptomic data from Leroch et al., 2013[15] and Blanco-Ulate et al., 2014[21] and the OG analysis from Voxeur et al., 2019[17]. The pectin lyase BcPNL1 was found to be the most expressed pectinase of the early development phase of the fungus (4-15 hour post inoculation) on plant-mimicking surface (A). PNLs can degrade fully methylesterified pectins to release unsaturated methylesterified OGs. This first degradation step leads to the production of long methylesterified OGs that can be further demethylesterified by BcPMEs and further degraded by BcPGs and probably BcPLLs. (B) At a later phase (2-3 days post inoculation) and when the fungus faces pectins with lower DM beyond the inoculation spot[14], BcPG1 is the most expressed pectinase. BcPGs can degrade partially methylesterified pectins and PNL derived OGs demethylesterified by BcPMEs to release unsaturated DP4 and unesterified DP3 OGs. These fragments can act as DAMPs to activate plant defense but can also be inactivated by oxidation through OG oxidases (AtOGOXs) to avoid excessive cell death and spreading of the fungus[26]. (C) Schematic representation of the enzymatic activity of pectin lyases and polygalacturonases on fully methylesterified HGA and demethylesterified HGA respectively.*

(A)

(B)

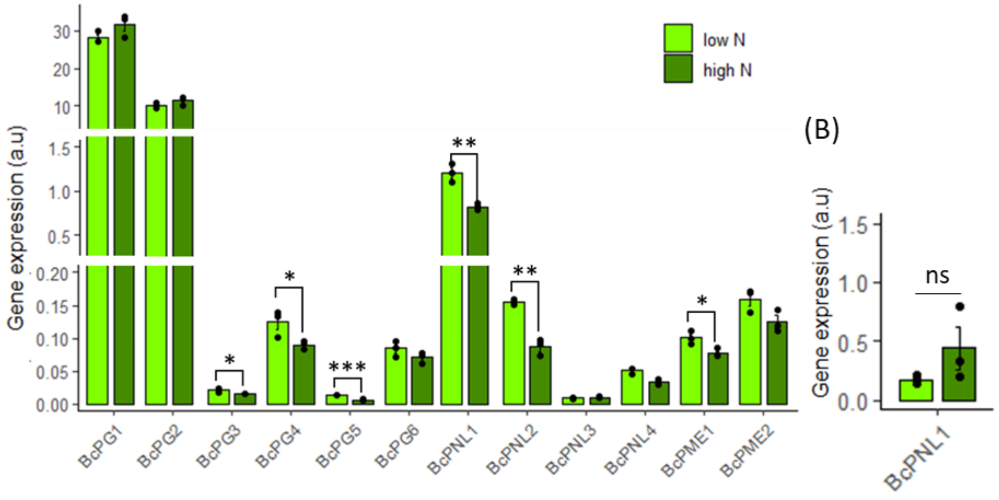

(C)

Common name	Identifier
BcPG1	Bcin14g00850
BcPG2	Bcin14g00610
BcPG3	Bcin04g04930
BcPG4	Bcin03g01680
BcPG5	Bcin01g07330
BcPG6	Bcin02g05860
BcPNL1	Bcin14g03430
BcPNL2	Bcin03g00280
BcPNL3	Bcin08g03820
BcPNL4	Bcin03g07360
BcPNL5	Bcin12g05620
BcPME1	Bcin08g02970
BcPME2	Bcin03g03830

Supplementary Figure 2: Impact of host nitrate nutrition on Botrytis cinerea pectinase genes expression. *(A) Pectinase genes transcript accumulation during infection in high and low N conditions at 1 day post-inoculation on detached leaves. (B) BcPNL1 expression at 15 hpi in liquid culture with detached leaves. Gene targets were normalized with BcACTA and similar results were obtained using BcUB1. Three independent experiments were conducted with similar results. Data are expressed as mean normalized expression in arbitrary units (a.u.) and are the means of triplicates (±SE). Statistical differences represented between high and low N are the results of a two sample t-test : *p<0.05, **p<0.01, ***p<0.001, ns: not significant. (C) Identifiers of studied fungal genes with their common names.*

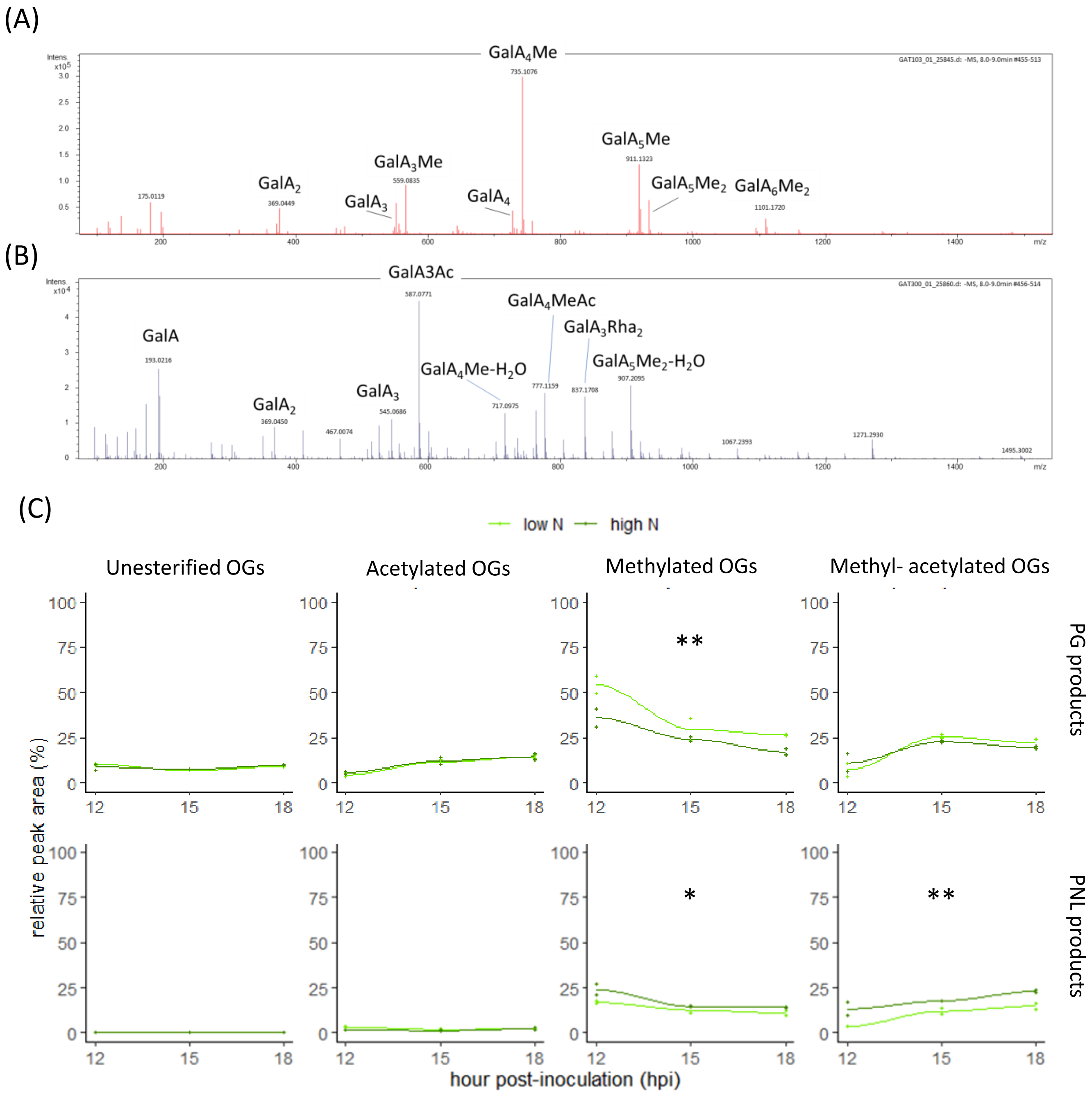

Supplementary Figure 3: The esterification status of the oligogalacturonides produced during Botrytis cinerea infection is impacted by host nitrate nutrition*. (A). Spectrum of the OGs produced by B. cinerea conidia incubated with citrus methylesterified pectins. (B). Spectrum of the OGs produced by B. cinerea conidia incubated wit acetyl- and methylesterified pectins originated from okra (C). LC-MS quantitative analysis of OG production after incubation of spores from the WT strain of the fungus for 12, 15 and 18 h with A. thaliana Col-0 leaves from plants cultivated at high or low N. The graphs represent the peak area of each group of products over the total of all detected OGs expressed as %. Dots represent the two independent replicates obtained for each time point. Statistical differences represented between the kinetics at high and low N are the results of a paired t-test: *p<0.05, **p<0.01.*

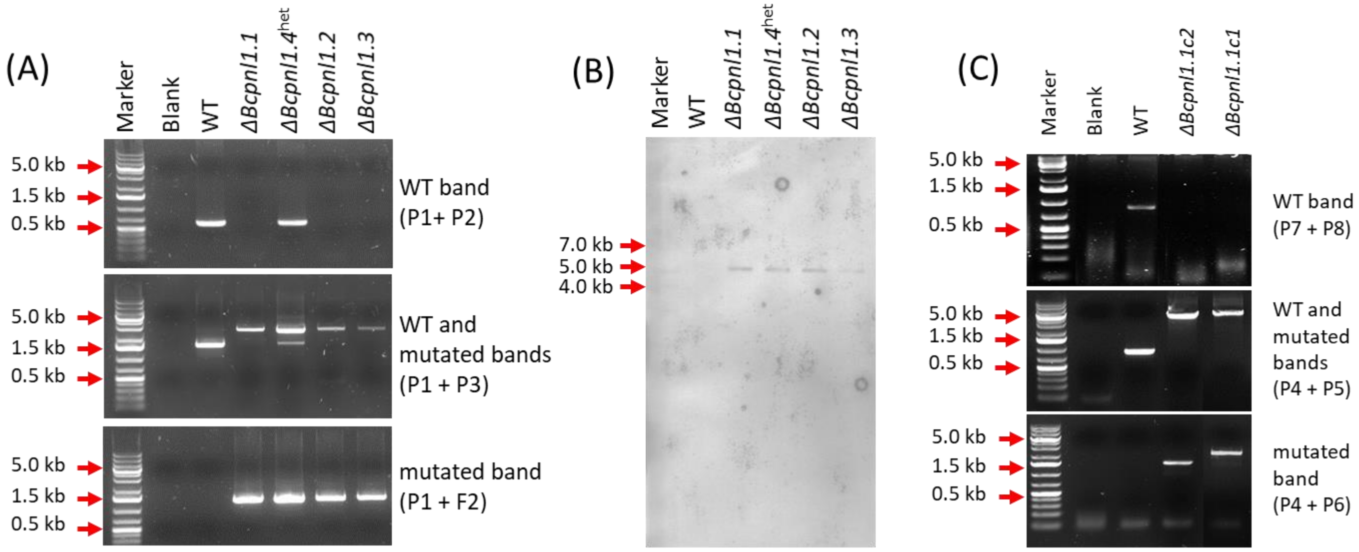

Supplementary Figure 4: Diagnostic PCR and Southern blot on the ΔBcpnl1 mutants and complemented strains. *(A) Diagnostic PCR showing successful integration of the fenR cassette and nuclear purity of the ΔBcpnl1.1, ΔBcpnl1.2 and ΔBcpnl1.3 mutants whereas ΔBcpnl1.4 is heterokaryotic. (B) Southern hybridation of genomic DNA. In order to detect multiple integration events, genomic DNA was digested by BglII and hybridized with a fenR cassette probe obtained by PCR with F1 and F2 primers. Only one band of the expected size was found in all transformants. (C) Diagnostic PCR showing successful integration of the complementation cassette and nuclear purity of the ΔBcpnl1.1c1 and ΔBcpnl1.1c2 strains. All primers used are described in Figure 2.*

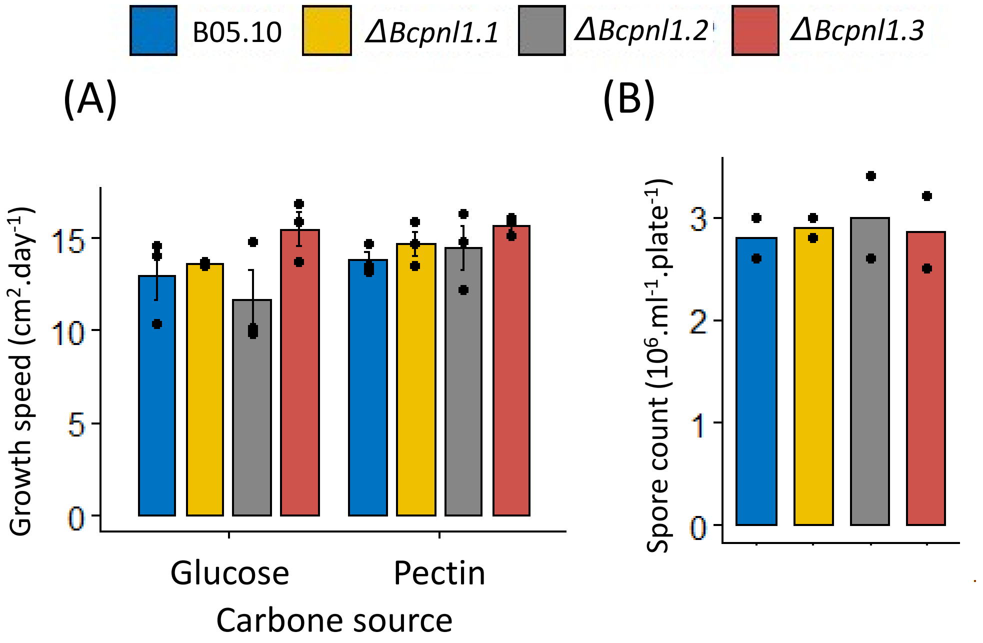

Supplementary Figure 5: Growth analysis of the ΔBcpnl1 mutants on different substrates*. In vitro linear growth speed with 1% glucose or pectin from citrus peel (DM>70%, Sigma-Aldrich) as carbon source (A) and spore production on PDA medium (B) are compared between B05.10 and the ΔBcpnl1 mutant strains. Data are the means of triplicates (±SE) (A) or duplicates (B).*

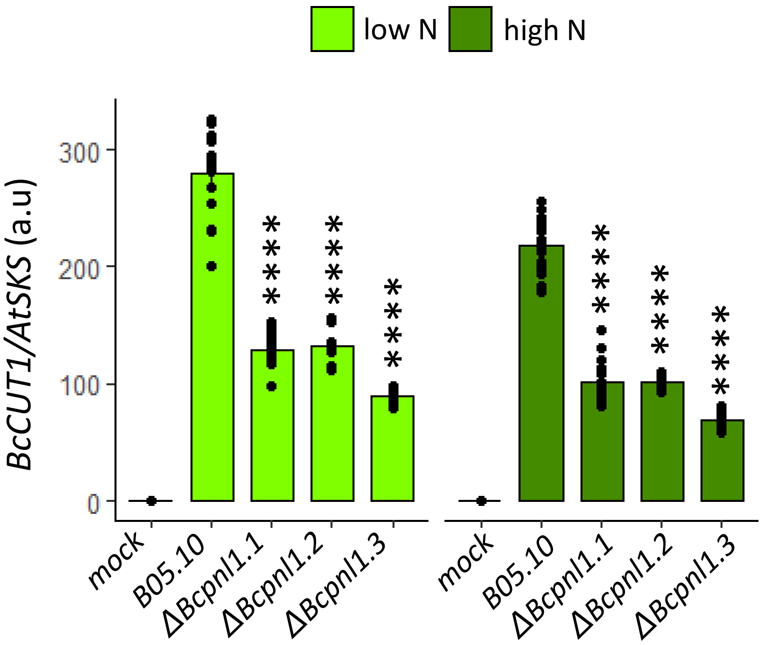

Supplementary Figure 6**: *Fungal DNA quantification of B05.10 and the ΔBcpnl1 mutant strains of B. cinerea during infection of A. thaliana detached leaves from Col-0 plants.*** *Data are expressed as mean (±SE) quantification of the fungal specific BcCUTINASE A (CUTA) gene over the plant specific AtSKS gene in arbitrary units (a.u.). Two sample t-test were conducted between the B05.10 strain and the mutant strains (C): ****p<0.0001.*

Supplementary Table 1: List of primers used to produce the strains studied in this work.

Primer name	Sequence	Use
P1	GGATGTTGAATGTCTCGAGGTT	Diagnostic PCR of the *ΔBcpnl1* mutants strains
P2	GAACCGAGCTTGATAGCACT	
P3	ATCTCACTCTCTCCTTCTGTTGT	
F1	AGATACCATTGCGCGAATCC	PCR amplification of probe for Southern-blotting
F2	TTCCTTGGGCTGCATTCAGT	PCR verification of the mutants/PCR amplification of probe for Southern-blotting
SgRNA1	AAGCTAATACGACTCACTATAGGTTGACGCCGCAGCAGTCATGTTTTAGAGCTAGAAATAGCAAG	Specific primer for SgRNA synthesis targetting *BcPNL1*
X1	AGTTATATCAATAACTTCACTTCAATCAATCATCATGGTTTCAATCAAGACCCTCGCTATCATTTTTTGGGCTTGGCTGG	Deletion cassette amplification
X2	CTTCATCCCTCCAGTCTACCATCTCCAACCCATCCTTAAATCTTTCCAATACCGGCGTTAGGCTCACTATACATCTGGC	
P4	GGCGTATAGCCATTAGTGTGC	Diagnostic PCR of the *ΔBcpnl1c* complemented strains
P5	TTACCAACGACTCTCTGCAGG	
P6	GAACCGAGCTTGATAGCACT	
P7	AGATCGCTGAGTCCATGATAGC	
P8	CACGAAGGGAATCTGGCTTG	
SgRNA2	AAGCTAATACGACTCACTATAGCGATGAAGATCTGAACCCCGGTTTTAGAGCTAGAAATAGCAAG	Specific primer for SgRNA synthesis targetting intergenic region on chromosome 3
X3	ACCCCACCACCATTGTGGTGTCTTCACTCGATGGAAATTTTACAGCGAGCTTGAGAATTGCCCGGAAAGGTGATCACAAGA	Complementation cassette amplification
X4	GCCCAAAAAATGCTCCTTCAATATCGGAAGTGAAGAGAGGCTACTGG	
X5	ACCCCACCACCATTGTGGTGTCTTCACTCGATGGAAATTTTACAGCGAGCTTGAGAATTGTGCAGCTGTGGAGCCGCATT	
X6	CGAGGGTCTTGATTGAAACCATGGTTGGATCGATTGTGATGTGATGG	
X7	CGTATGTAGATAAGATGTATGATTAGGGG	
X8	GCCCAAAAAATGCTCCTTCAATATCATCTTGTTGGGGGGAAGG	
X9	ATGGTTTCAATCAAGACCCTCG	
X10	CCCCTAATCATACATCTTATCTACATACGCACTTAAATCTTTCCAATACCGGCG	
X11	GATATTGAAGGAGCATTTTTTGGGC	
X12	CCCTAGAAACATCTACAAATCAACGTCTCAAACGATGAAACCCCACGCCATCCATGCAGCCTCACTGATACATCTGGCACCTAC	
X13	ACCCCACCACCATTGTGG	
X14	CCCTAGAAACATCTACAAATCAACG	

Supplementry table 2. List of primers used to for RT-qPCR on fungal genes.

Gene name	identifier	Primer orientation	Sequence
BcACTA	Bcin16g02020	fwd	CGCCATTGCTCGTGTTGAC
		rev	TCGGCAGTGGTGGAGAAAGT
BcUBI	Bcin11g03430	fwd	ACCATAACAACTCGGCATGC
		rev	ACCGCGATGTAAAACTGACG
BcPME1	Bcin08g02970	fwd	TATCTCTGCCCACACCAC
		rev	CGACGACGAGACATTTA
BcPME2	Bcin03g03830	fwd	AAAACAACGAAGCCACCCTC
		rev	CGTAGGAGGAGAGAGCAAGG
BcPG1	Bcin14g00850	fwd	CCCTCTCCGGCATTACATCC
		rev	CGTTGGTAGCACTGGAGGAG
BcPG2	Bcin14g00610	fwd	TCAAAACCGGCACCAAAGTT
		rev	CCAAGTCCATCCCACCATCT
BcPG3	Bcin04g04930	fwd	CTCAGCTTCCACTGGTCTCC
		rev	GCAACAGTAGCCAAGGTGGT
BcPG4	Bcin03g01680	fwd	GCCCACGACTTGACTGATTC
		rev	TTGCCCAAAGAATCACCAGC
BcPG5	Bcin01g07330	fwd	CGACCTTGGATTTGACAGGT
		rev	GAGACCAAAGGACCAGACCA
BcPG6	Bcin02g05860	fwd	GCCCGTCCATCTTTTCACTA
		rev	CTGGACGAGCTGACATCAAA
BcPNL1	Bcin14g03430	fwd	AGCTTGTCAACTGGCTCTCC
		rev	GTTGGAGATGGTGACACGGT
BcPNL2	Bcin03g00280	fwd	TCGTGTCATCGTTCTCACCA
		rev	TGGAACCCTCACCGATCAAA
BcPNL3	Bcin08g03820	fwd	CAACCAGGCTCTTCGATTCG
		rev	CACAATGTGTTTCCTGCCGA
BcPNL4	Bcin03g07360	fwd	AGCTGGCAGACTCACAATCT
		rev	GGCCTGCGACATTGTAGAAG
BcPNL5	Bcin12g05620	fwd	AGCCGTCTAATGGTGGTCAA
		rev	CCAAACATGTGCAGGGTTCA

Supplementry table 3: List of primers used to for RT-qPCR on plant genes.

Gene name	identifier	Primer orientation	Sequence
AtAPT1	AT1G27450	fwd	CCTATTGCGTTGGCTATTG
		rev	TCTTCACTCCTACTCGTTC
AtUBI4	AT5G20620	fwd	TGACACCATCGACAACGTGA
		rev	GAGGGTGGACTCCTTCTGGA
AtPDF1.2a	AT5G20620	fwd	TTTGCTTCCATCATCACCCTTATCTT
		rev	ACACTTGTGTGCTGGGAAGA
AtPR1	AT2G14610	fwd	CTGGCTATTCTCGATTTTTAATCG
		rev	TCCTGCATATGATGCTCCTTATTG
AtPR2	AT3G57260	fwd	AGTCGGGACGAGTGTGGAAA
		rev	GCCTTCTCGGTGATCCATTC
AtPAD3	AT3G26830	fwd	CCTCGTCCTCTTACCCCTGA
		rev	GAAGCTTCTTTGGACCCGGA
AtWRKY70	AT3G56400	fwd	CCCAAGAAGTTACTTTAGATGCAC
		rev	TTGCTCTTGGGAGTTTCTGC
AtJOX3	AT3G55970	fwd	GAACCAGCTCCTCATGCTTT
		rev	GGGTTCACGATCACTCTGTG
AtJAZ1	AT1G19180	fwd	ATGTCGAGTTCTATGGAAATGTTCTG
		rev	TCATATTTCAGCTGCTAAACCGAG
AtJAZ3	AT3G17860	fwd	ATGGAGAGAGATTTTCTCGGGTTGG
		rev	TTAGGTTGCAGAGCTGAGAGAAGAAC
AtJAZ4	AT1G48500	fwd	ATGGAGAGAGATTTTCTCGGGCTG
		rev	TTAGTGCAGATGATGAGCTGGAGG
